# Hospital and Institutional News

**Published:** 1919-04-12

**Authors:** 


### HOSPITAL AND INSTITUTIONAL NEWS.

### POST-GRADUATE TEACHING.

The scheme for the establishment of a Post-Graduation Association in London was recently approved at a meeting of the representatives of post-graduate schools and special hospital. Organising committees have been appointed, and a public meeting will probably be held at the Royal Society of Medicine on the afternoon of April 29, at which the President of the Board of Education will, it is hoped, take the chair. It is impossible here to give details of the proposed arrangements. They can be found in full in contemporary medical journals, and we must be content with expressing our approval of a most comprehensive scheme. Not only are the requirements of the medical man met, but many alternatives of time and opportunity are provided. The principle of central organisation is well to the fore, and the financial side of the scheme appears satisfactory. The income of the Association would be derived from interest of the endowment fund, registration fees of students, fees for courses, and Government funds. There is no time like the present for bringing such a system of teaching into being, and one hopes that it will, either in its present or some very slightly modified form, meet with immediate and general acceptance.

### THE LATE SIR W. CROOKES.

The death of this well-known pioneer of research will cause the deepest regret among his contemporaries and followers in every scientific profession. The value of his contributions to knowledge and their indirect bearing upon medical work should be fully appreciated. Probably we have not, in this branch, touched more than the fringe of the benefit ultimately to be derived from his discoveries and the inestimable worth of so keen and fertile an intellect, which could show equal activity in wonderful electrical inventions, the electronic theory, the discovery of many new elements, the problem of the world's wheat supply, and the fixation of nitrogen from the air, will not be fully brought home to us until time has allowed of fuller application for that great number of physical and chemical factors he has wholly or partially discovered. In later days his interest in spiritualism was well known. In 1896-99 he served as President of the Society for Psychical Research, and twenty years previously had published the well-known "Researches on the Phenomena of Spiritualism." The Royal Society, of which he was elected Fellow in 1863, awarded him three of the medals at its disposal—a Royal Medal in 1875, the Davy Medal in 1888, and the Copley Medal in 1904; appointed him to deliver the Bakerian Lecture on three occasions; and finally chose him as President. He held a great number of foreign decorations, and with his death Great Britain loses yet another of that famous school of pioneers in science of which at the close of the nineteenth century she was so justifiably proud.

### CHILDREN'S HOSPITAL FOR BERMONDSEY.

Princess Marie Louise has consented to a proposal submitted by leading residents of Bermondsey that the Princess Club Hospital for Wounded Soldiers, in Jamaica Road, shall be converted into a hospital for children when the Army Council no longer require it. The hospital was originally a club for factory girls, but since the outbreak of war has been a hospital for wounded and sick soldiers. About 2,300 patients have been nursed here, mainly men belonging to Bermondsey. As Her Highness points out in a letter, no more appropriate war memorial, could be erected, as the hospital would care for the children of many of those who have been nursed back to life and health within its walls during the war. Dr. King Brown, Medical Officer of Health for Bermondsey, has taken a prominent part in inducing the Princess to consent to the scheme. The hospital is an ideal one for children, and is equipped with modern appliances. It is to contain 50 beds in two upper wards, whilst the ground floor will be utilised for the out-patients' department. Dr. Brown hopes to have eye, dental, and orthopædic departments. The only objection to the scheme raised so far is that the administrative block is old. It comprises six old houses, but these could be rebuilt with a new frontage that would add dignity to the hospital. It is to be hoped that the necessary funds will be raised, for Bermondsey, with its huge child population, is badly in need of such a hospital.

### AN INTERNATIONAL HEALTH BOARD.

At the recent conference of the Red Cross Societies of the Allied Nations at Cannes, Colonel Richard Strong, Director of the Department of Medical Research at Harvard University, gave news of a scheme practically amounting to the provision of an international board of health. The enormous advantage of preventive action over attempts at relief was emphasised on all sides and with regard to every disease of the war period. Therefore the proposed International Red Cross Bureau is taking this line and should find unlimited scope for work in many lands. There is a marked distinction to be drawn between those countries where good official and voluntary preventive work already prevails, and those where chance still retains the upper hand. In the former invaluable societies were already at work, and the new Bureau was intended neither to supercede nor interfere with them. Its function was to originate, organise, and launch the new methods and the new spirit into lands where the need was greatest, and when one centre grew to self-sustaining power, pass on to the establishment of the next neglected area. We have but a passing report, but the news is of great promise.

### TWILIGHT SLEEP.

We are in receipt of a pamphlet emanating from the Bushey Lodge movement to establish Twilight Sleep Maternity Homes throughout Great

Britain, and urging the Governmental and general adoption of this method of, wholly or partially, painless child-birth. A short time ago was published in this journal an attempt to provide explanation of the exact position of this mode of treatment in relation to other medical procedures of the day, to count the risks and to state the essential requirements. It was then described as a method which called for not only the very highest technical skill, but also the ideal conditions of a hospital before a justifiably high percentage success could be expected. Promoters of this scheme undoubtedly have the full extent and meaning of such necessities in view, and will urge not only the establishment of the centres, but the provision or development of a large and highly trained staff to administer the actual treatment. In its ideal form this project is fully deserving of attention, but it must be remembered that a national organisation of twilight sleep centres raises many more vital and serious questions of efficiency than similar widespread establishment of most forms of medical institution. It is by no means a venture to be lightly undertaken, and to us appears to admit of many objections.

### VENEREAL INFECTION.

In the past week there has appeared yet another letter in the *Times* urging more rational treatment of this menace, and on this occasion reference is made to the possibility of compulsory notification without undue publicity. America has proved it can be accomplished, and we have already given several examples of her success in previous issues of this journal. Many States have systems in efficient working order, and the good results are already becoming obvious. It has been to us a matter of great surprise that the British authorities have been so backward to foresee the success of the strict methods for a deadly disease, and we have previously attempted to show that our health advisers seemed loth to view the situation from a detached and unprejudiced standpoint. Modern conditions call for modern ways, modern "education" in these matters, even among the relatively young, is undisguisedly widespread, and we can do little good by a pretence at secrecy and modesty. The profession need strictness, tact, and sympathetic understanding, but must have the force of law to back it in preventive endeavours. We hear that a notification scheme has at last been propounded, and may shortly appear for official confirmation. We sincerely hope that it will be modelled on the firm lines taken by those responsible in America.

### ENFIELD COTTAGE HOSPITAL'S LOSS.

The Annual Meeting of Governors which was to have taken place on the 29th March was postponed owing to the death of the Honorary Secretary, Mr. James Penny, which occurred with tragic suddenness on the morning of that day. Mr. Penny called at the printers for some advance copies of the annual report, and, whilst waiting in the office, he was seized with a cerebral attack which terminated fatally in a few minutes. Mr. Penny held the post of Honorary Secretary to the Hospital for twenty-eight years, and had devoted himself to its welfare with solicitude and indefatigable industry. The recent conflict with the medical staff was a source of considerable anxiety to him, and his impending resignation under the circumstances arising therefrom had occasioned him keen regret. Mr. Penny was universally esteemed, and in the view of those most intimately acquainted with his work the loss which the hospital has sustained by his death cannot be adequately expressed.

### THE APPOINTMENT OF PUBLIC DENTISTS.

The Medical Officer of Health for Hertfordshire has good reason for the blessing which he gives to the Watford Dental Treatment Centre. In 1918 the number of children treated rose to 456, practically twice as many as in the previous year, and all this good work was accomplished by a voluntary agency. Its example is proving infectious, for the County Council is preparing a scheme by way of experiment; but this scheme will need much supplementing by the Dental Treatment Centre. Already it has enough work "for a whole-time dentist for six days a week, and the appointment of such an official," says the medical officer of health, "should not be delayed an hour longer than is necessary." Meantime, as always, it is for the voluntary system to show the way. Was it not odd that the late Mr. Kirkman Gray, who wrote a history of such pioneer work in the nineteenth century, used to describe this familiar process as "the failure of philanthropy"? Infancy is a failure in the same sense, and so the voluntary system in Watford, as elsewhere, should pursue its pioneer work undeterred by the ingratitude of Fabian-like historians.

### DEMOBILISATION OF WAR CHARITIES.

The Charity Commissioners have issued circulars to registration authorities pointing out that war charities whose activities have ceased since the armistice was signed should apply for authority to use their unapplied funds for objects approximating to their original purpose. The Commissioners take the view that these funds are held upon charitable trusts. The British Red Cross Society and Order of St. John of Jerusalem have an Act of Parliament (1918) of their own, which was framed in consultation with the Commission, and application of surplus funds is regulated by this Act. During 1918 legal proceedings were taken against seventeen persons appealing on behalf of unregistered war charities, and ten were convicted. The Commission's experience of the working of the Act during the years 1917 and 1918 has confirmed their view that further legislation, presumably for the general registration of charities, is necessary. It does not appear probable at first sight that respectable charities would raise any objection to this project. Already, through the Local Government Board, a system of registration in respect of charities for the blind has been commenced.

### THE FUTURE OF DENTAL TEACHING.

Manchester Dental Hospital, like most dental hospitals, needs enlargement, and, to strengthen its connection with the University, is to provide a post graduation course. The students include the women, and now that the war has proved how prevalent is dental caries, the demand for dentists is going greatly to increase. The trouble is that few can afford the fees. But since universities, like Manchester University, need to improve their medical course, the solution is close in the dependence between dental hospitals and universities, so as to make the profession accessible to educated men and women at the former and to attract the new students to the universities, which must cater for them by much improving the dental course. This can be accomplished only by affiliating the dental hospitals to the universities.

### THE GOVERNMENT AND ST. GEORGE'S HOSPITAL.

Somewhat unnecessary consternation seems to have been caused at Malton by a statement made by Major Behrens which purported to forecast the Government's intentions in regard to the voluntary hospitals. Dr. Addison being ill, Sir Auckland Geddes was interviewed, at Major Behrens' instance, by Mr. E. R. Turton, M.P. Sir Auckland Geddes told him, said Major Behrens, that although the Government Bill was somewhat vague, yet it was "certain that the Government would be taking over St. George's Hospital and other hospitals in the country within the next forty years." Major Astor has since stated that there is no foundation for this suggestion.

### THE PRINCE OF WALES AT BETHLEM.

A private visit was paid last week by the Prince of Wales to Bethlem Hospital, that veteran institution whose history is as long as the publicity its work now receives is little. Dr. Porter Phillips, the medical superintendent, Lt.-Col. H. L. McCarthy, D.S.O., the assistant medical officer, and Mr. J. L. Worsfold, the clerk, received the Prince, who made a tour of the institution and inquired into the percentage of recoveries, and seemed impressed by the cheerfulness of most of the patients. In Dr. Johnston's time, that was not the impression carried away by visitors, who went to Bedlam, and Bridewell, much as people now go to the Zoo. How Puice and his teaching changed the fetters into fellow-feeling, and primitive methods into scientific treatment, in which recreation, dances, and concerts have their important share, is now an old story. But, while embracing the change, Bethlem still remains one of the historic monuments of London.

### VOLUNTARY HOSPITAL DISPENSERS.

Mr. F. A. Hocking, Chief Pharmacist, London Hospital, with several other pharmacists, issued an invitation to voluntary hospital dispensers to meet on April 9 to consider the question of the formation in the Incorporated Association of a section for dispensers. The object is to form a committee to advise the Council on matters affecting dispensers. Already there are two organisations representing hospital pharmacists—the Public Pharmacists' Association and the Chemists' Union, Public Services Section—but membership is not confined to voluntary hospital pharmacists alone. The idea of the proposed "section" is to confine it to voluntary hospital dispensers, which, of course, will include all dispensers whether they hold a pharmacist's qualification or other qualification in dispensing. A condition, of course, will be that prospective members are already, or intend to become, members of the Incorporated Association of Hospital Officers.

### TWO SANITARY ADMINISTRATIVE MATTERS.

There is to be a special Board of Health for Wales, while school medical officers are to be removed from the ægis of the Board of Education and transferred to the Ministry of Health. Thus decentralisation apparently triumphs in the first case and loses in the second. So very much depends, however, on the details of practical administration that it is hard to draw any working conclusion as to what has really happened. In sanitary medical services centralisation means actually a purer and more efficient administration at some little risk of friction and offending local susceptibilities. One type of mind will always consider this a gain; the other will always think it a loss. That is really all there is to be said on the matter.

### INQUIRY INTO INDUSTRIAL TUBERCULOSIS.

Under the auspices of the Medical Research Committee a report on industrial tuberculosis has been drawn up by Mr. Greenwood and Dr. Tebb, respectively of the Lister Institute of Preventive Medicine and the Ministry of Munitions, Welfare Section. The method used is mainly statistical, and the main conclusion reached is certainly to convict industrialism—even, as is evident from the date of some of the material, the most modern and improved form of it—of being an agent in producing tuberculosis. The particular point first commented on by the Registrar-General in his seventy-ninth annual report—increase of tuberculosis in young female munition workers—receives support. Among women there is a nearly uniform increase of the proportionate phthisis mortality at ages fifteen to twenty in the great industrial towns. This had also been pointed out by the medical officers of health for these areas. But the female death-rate at working ages has now for years been so much below the male that this is a phenomenon not so serious as might be imagined.

### WHY MUST MASTER AND MATRON BE MARRIED?

The resignation of the master and matron of the Pickering Workhouse, Yorkshire, raises an old institutional question not yet solved. It is proposed to appoint Miss Lawes, the assistant matron, to the matronship. But she is, as the prefix explains, unmarried, and her colleague will have to be either the relieving officer, a possible applicant, or a bachelor, if the assistant matron fills the vacant post. Though the practice is in favour of appointing a man and his wife to these joint positions, there are precedents on the other side. No one has yet suggested that the proposed new chief officers intend to marry. That would be the easiest solution, administratively. This practice of having married administrators for institutions which are not limited to patients of one sex is confined, we believe, to workhouses. Why should it be more necessary in them than elsewhere? Is it because the duties of the twain are ill-defined? Or because a married couple is cheapest to the Guardians, which means that masters and matrons cannot afford to marry (in other words, cannot afford a decent life) on the income of their individual work? Or is it because the type of person to whom they and their protégés belong are particularly obnoxious to scandalmongers? Since we have long pilloried the condition "no encumbrances" in advertisements for married applicants for these posts, the answer to these questions would seem to be the economic one. If so, it is hardly less disgraceful than the assumption suggested by question four.

### DONATIONS NOT RECOVERABLE.

Judge Cluer, at the Shoreditch County Court, was called upon to decide whether a donation of ten guineas to the Metropolitan Hospital could be recovered in an action for damages. The plaintiff, whilst crossing Whitehall, was knocked down by a taxi-cab, sustaining two fractured ribs. He was first treated at the Charing Cross Hospital, and later was a patient at the Metropolitan Hospital for a month. When he left the latter hospital he was so pleased with the treatment he had received that he made a donation of ten guineas to the funds. In bringing an action against the owner of the taxi-cab for damages he asked that this donation should be included, but it was argued that it could not be recovered. If a man had a bad illness, so counsel contended, and went on a friend's yacht for a trip, and gave a good tip to the servants because they had treated him so well, he could not recover it if anything happened. It was a gratuity or tip, given out of goodness of heart—free. Judge Cluer agreed with this contention, and held that a donation to a hospital was not recoverable as damages. The decision seems not only good law, but common sense. A contrary view might leave the door open to donors to indulge in second thoughts.

### MANSFIELD TRADERS REBUKED.

There was plain speaking at the annual meeting of the Mansfield and District Hospital, Mr. J. P. Houfton, managing director of the Bolsover Colliery Company, pointing out that local tradesmen had failed to realise their responsibility to the institution. Although they have never done so well as during the last three years, their response to an appeal for forty thousand half-crowns to clear off the deficit resulted in only thirteen thousand being received. This result was contrasted with that at Chesterfield, where the tradesmen last year raised £4,500 to wipe out the deficiency on the hospital and another £4,000 for expansion. Once more the miners in the Mansfield area have shown a fine example. Colliery workmen's subscriptions last year amounted to £2,036, as compared with £806 from colliery companies, and £710 in subscriptions from other sources.

### A TRUST DEED AND HOSPITAL SERVICES.

The Church of England, in the person of Canon Newton, has set an example to other denominations in connection with the chaplaincy of Smallwood Hospital, Redditch. On being asked why chaplains of other denominations were not free to hold services in the hospital, the hon. secretary, Mr. W. Baylis, referred to a by-law in the trust deed which runs as follows: "The spiritual care of the patients shall be under the immediate control of a chaplain, who shall be a clergyman of the Church of England. Other clergymen of the Church of England and ministers of other denominations shall, at the request, or with the consent of, any patient be free to visit that patient, but must confine their instructions and ministrations to that patient alone." After discussion, the chairman, Mr. Charles Terry, said that he did not think there would be any objection if the Nonconformists wanted to hold a service, as Mr. Baker proposed, and appointed one of their number to represent them. The ministers could look in to see if there were any patients of their own denominations. Mr. Baylis said that in practice, ministers could come at any time. It was for the chaplain, Canon Newton, to decide about the holding of services. Canon Newton replied that, because week-day services were difficult to arrange, he held one at a specified time on Sundays, and was limited to three-quarters of an hour. So far as he was concerned, he would not mind any one holding services there. In sum, if members of any denomination are patients in the hospital, there is nothing in the trust deed to prevent a service being held, provided that the service is confined to the members of the denomination whose minister holds it. Canon Newton has acted in accordance with the best traditions of the Church of England.

### THIS WEEK'S DRUG MARKET.

With the near approach of the Easter holidays business is naturally quiet. The downward tendency of prices is still the chief feature of the market. One important change is a fall in the price of quicksilver, which has been followed by a reduction in quotations of calomel and other salts of mercury. Methylated spirit has been reduced in price, as also have methylated ethers. The downward tendency in the price of permanganate of potash continues, but there is a long way to go before pre-war values are reached. One of the few articles which are dearer is cocoa butter; menthol and American peppermint oil are also maintained at high prices. With further arrivals of eucalyptus oil of good quality, a brake is kept on any advance in price that might result from the continued good demand. Honey is cheaper. Most of the synthetic drugs continue their downward price tendency. Cascara sagrada tends downwards in price. The price of camphor is firmly maintained. Ergot of rye continues very scarce and dear. Magnesia is more plentiful and lower in value. Liquid paraffin tends downwards. Sugar of milk is rather cheaper.

